# The analysis of genetic variation in the mitochondrial genome and its application for the identification of *Papilio* species

**DOI:** 10.1080/23802359.2018.1481776

**Published:** 2018-06-18

**Authors:** Zuo Ruihua, Jiang Ping, Sun Chuanbo, She Deyong, Zhang Feng, Hu Chaochao

**Affiliations:** a College of Biological and Pharmaceutical Engineering, West Anhui University, Lu’an, China;; b Animal Healthy Breeding and Animal Epidemic Monitoring and Warning Enter, West Anhui University, Lu’an, China;; c College of Fisheries and Life Science, Shanghai Ocean University, Shanghai, China;; d Analytical and Testing Center, Nanjing Normal University, Nanjing, China

**Keywords:** *Papilio*, mitochondrial genome, genetic variation, mean clock rate, phylogeny

## Abstract

Mitochondrial DNA (mtDNA) markers are ideal for evolutionary studies, including phylogeography, population genetics, phylogeny, etc. However, different mitochondrial genes always own different evolutionary rate. In this study, we analysed the genetic variation across the 16 complete mtDNA from 13 species in the genus *Papilio* and recognized the best DNA barcoding for *Papilio* species. The mitochondrial gene arrangement for each species shares a similar order, similar to the typical Papilionidae species, which indicated the relatively conservative state of gene arrangement in *Papilio*. The sliding window of genetic diversity showed that there was a significant difference in the genetic diversity of each gene in the mitochondrial genome of *Papilio*. The relatively mean clock rate of the *ND1* was broadly lower than the other genes in mitochondrial genome of *Papilio*; while the ATP8 owns the largest values of mean clock rate. Those results suggested that the rate of evolution of each gene is not balanced and all mitochondrial genes except *ND1* and ATP8 could act as barcoding for the identification of *Papilio* species. The phylogenetic analyses of complete mtDNA data for 13 Papilio species divided them into five major branches, which keep the same topological structure with previous studies.

## Introduction

The *Papilio* is a genus in the swallow tail butterfly family Papilionidae (Vattikonda et al. 2014). As a widespread species all around the world, it includes a number of well-known species, such as *P. polytes*, *P. polymnestor*, *P. memnon*, and *P. deiphobus* (Sbordoni and Forestiero 1998; Condamine et al. 2013; Cong et al. 2015). Nowadays, about 210 *Papilio* species have been documented worldwide, with some 27 species recorded in China (Wu 2001). As they are highly sensitive to habitat loss and fragmentation, pollution and climate change, the genus face the great crisis of survival (Settele et al. 2009; Li et al. 2011). In IUCN, about 12 species in this genus are considered as vulnerable or endangered species, which make more urgent to investigate the genus. In addition, the swallowtail butterflies were also acted as model organisms for studies in evolutionary biology and conservation biology (Maes and Dyck 2005; Zhu et al. 2011).

The typical metazoan mitochondrial DNA (mtDNA) is a double-stranded circular DNA molecule, which encodes a conserved set of 37 genes, including 13 protein-coding genes (PCGs) plus the two ribosomal RNA (rRNA) genes and 22 transfer RNA (tRNA) genes (Shadel and Clayton 1993; Boore 1999). This, together with other special characteristics such as faster evolutionary rates than nuclear genes, presumed maternal inheritance, and absence of recombination makes mtDNAs one of the most popular targets for population genetics studies and accurate identification and differentiation of a cryptic species (Zakharov et al. 2004; Kim et al. 2005).

Herein, in this study, we clarified the 16 complete mitochondrial genome sequences about 13 *Papilio* species in China, collected from NCBI, and compared these sequences and genetic variation with each other. In addition, with the help of online software OGDRAW (http://ogdraw.mpimp-golm.mpg.de/) to create the mtDNA ring structure, calculate the relative mean clock rate of each gene and also the phylogenetic relationships of this genus using mitochondrion sequence datasets.

## Results

### Genome organization

**Figure 1.**
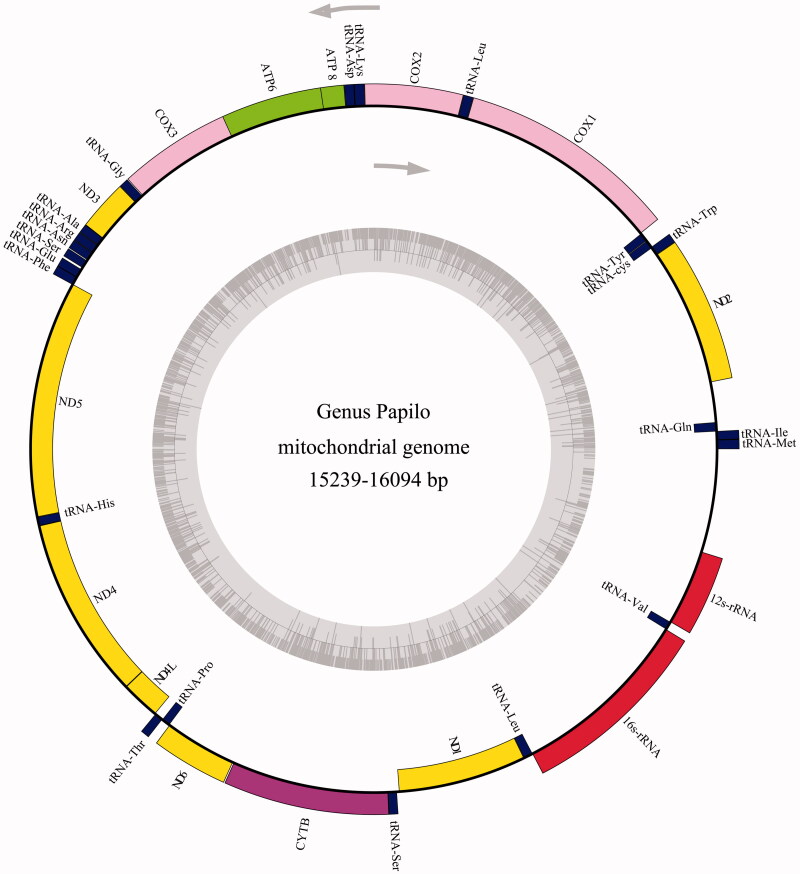
Gene map of the *Papilio* mitochondrial genome. Genes lying outside of the outer circle are transcribed in the clockwise direction whereas genes inside are transcribed in the counter clockwise direction.

**Figure 2.**
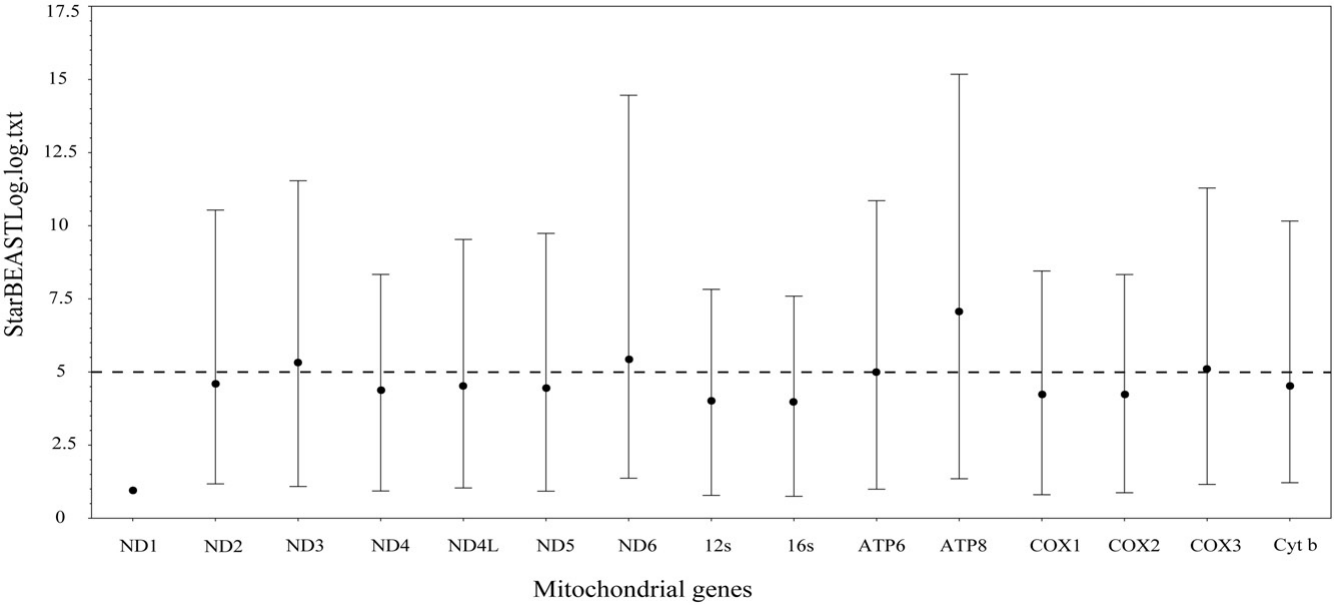
The comparison of relatively mean clock rate of 15 mito genes. Bars indicated the 95% CI.

The complete mitochondrial genomes of the species in the genus *Papilio* were from 15,239 bp to 16,094 bp (Table S1). The organization of the sequence encodes 13 protein genes (*ATP6*, *ATP8*, *COI*–*III*, *ND1–6*, *ND4L*, and *Cyt b*), 22 tRNA gene and a putative control region (*D-loop*) (Figure 1). Among these PCGs, the longest one is *ND5* genes (1719–1740 bp) which is located between the *tRNA^Phe^* (64–70 bp) and *tRNA^His^* (64–69 bp), the shortest one is *ATP8* gene (165–171 bp) which is located between tRNA^Asp^ (64–68 bp) and *ATP6*. Some PCGs like *ND2*, *ATP8*, and *ND3* usually start with an ATT codon, and for most species, *COII*, *ATP6*, *COIII*, *ND6*, and *Cyt b* usually consider ATG as their start codon (Table S2). Besides, the codons CGA, TTA, and CTA are often present in the *COI*, *ND4*, *ND4L*, *ND5*, and *ND1*. TAA is the most frequent stop codon in this genus, besides, the *ND1* and *ND3* end with TAG. About 22 tRNA genes contain the mitochondrial genome, the length of them are ranges from 60 to 70 bp. The sliding window of genetic diversity results shows it has a significant difference in the genetic diversity of each gene in the mitochondrial genome of *Papilio* (Figure S1). Relative low genetic diversity was found in the mtDNA *16S* gene compared with those of the other genes; the mtDNA *ATP8* gene owns the relative high genetic diversity (Table S3). Comparison of 15 genes revealed that the relatively mean clock rate of the *ND1* was broadly lower than the other genes in mitochondrial genome of Papilio; while the *ATP8* owns the largest values of mean clock rate (Figure 2, Table S4).

### Phylogenetic tree

The BI and ML phylogenetic trees, which are based on the complete mitochondrial genome among 17 *Papilio* species have the same topology (Figure S2). The analysed species are divided into five major clades. The clade A makes up the first lineage, which is sister to the second group, clade B. The clade C forms the third group and is sister to the clades A and B. The clade D presents the same topological structure with clade C. The lineage consisting of these four groups in turn is sister to the fifth clade, clade E.

## Discussion

### Mitochondrial genome annotation and features

As a huge genus, the *Papilio* owns about 210 species all over the world (Wu 2001). However, habitat loss and fragmentation are regarded as the greatest threats to global biodiversity and about 12 species in this genus become vulnerable or endangered species which is recorded in IUCN. For most species in the order of Lepidoptera, the mitochondrial genomes appear as the same topological structure and arrangement, which has no introns, no long intergenic spacers with only a few overlapping sequences. In this study, we compared the length of the 13 species in Papilio, and found the length of them is similar with each other. The longest of one from them is *P. maraho* (16,082 bp) and the shortest one is *P. machaon* (15,185 bp). The overall base composition is: A, 38.9–40.5%; T, 40.0–41.6%; C, 11.3–12.8% and G, 7.2–7.9%. The A + T content is higher than G + C, the detailed information is listed in Table S2. Compared with the mitochondrial genomes in the 13 species, the A + T compositions of *Papilio* mtDNA are similar, and it shares with the other genomes a strong AT bias (Table S3). Guanine (G) is the rarest nucleotide; the percentage of the other three bases is roughly equal to each other, similar to other Lepidoptera animals. GC and AT skews are a measure of compositional asymmetry; in arthropods mtDNA, GC–skew values are all negative (G < C), and the AT–skew is also negative (A < T). In *Papilio* mtDNA, GC–skew (0.18–0.26) and AT–skew (0.01–0.03) values are in accord with this principle (Table S3).

### DNA barcoding

DNA barcoding has potential to identify species because sequence divergences are ordinarily much lower among individuals of a species than between closely related species (Hebert et al. 2003, 2004; Song et al. 2008). For establishing DNA barcoding for species, the orthology of characters is the fundamental factor. The ideal DNA barcoding should have an observable gap between intra- and interspecific levels of divergence and correctly identify species (Hebert et al. 2004; Meyer and Paulay 2005). Previously, the region of *COI* gene is recommend as the universal barcoding marker for many animal groups (Hebert et al. 2004; Ward et al. 2005; Ratnasingham and Hebert 2007). However, if the orthology assumption is violated, that is, whether paralogous sequences are unknowingly treated as orthologs, incorrect inferences barcoding are inevitable (Funk and Omland 2003). For example, a number of molecular evolutionary processes can hinder correct amplification and identification of the orthologs (Rubinoff et al. 2006), including duplication of the gene of interest within the mitochondrial genome (Campbell and Barker 1999), nuclear integration of mtDNA (Bensasson et al. 2001), and heteroplasmy (Frey and Frey 2004). For avoiding the incorrect inferences barcoding, multiple genetic marker may be helpful. Therefore, variety of genetic markers, own the similar genetic substitution rate, may be needed. In our study, the sliding window of genetic diversity showed that there was a significant difference in the genetic diversity of each gene in the mitochondrial genome of *Papilio*. In addition, the relatively mean clock rate results suggested that the rate of evolution of each gene is not balanced and all mitochondrial genes except *ND1* and *ATP8* could act as barcoding for the identification of *Papilio* species. These results showed that the relatively mean clock rate of the *ND1* was broadly lower than those of the *COI* (about 3.81 times), *12S* (about 3.55 times), *16S* (about 3.58 times), and *COII* (about 3.98 times) genes. In conclusion, the identification of *Papilio* species may be selected two or more genetic markers, such as the combined of *COI* and *12S* genes, which was helpful to avoid the incorrect inferences barcoding.
